# Mathematical modeling and stability analysis of macrophage activation in left ventricular remodeling post-myocardial infarction

**DOI:** 10.1186/1471-2164-13-S6-S21

**Published:** 2012-10-26

**Authors:** Yunji Wang, Tianyi Yang, Yonggang Ma, Ganesh V Halade, Jianqiu Zhang, Merry L Lindsey, Yu-Fang Jin

**Affiliations:** 1 Department of Electrical and Computer Engineering, University of Texas at San Antonio, San Antonio, USA; 2 Department of Medicine, University of Texas Health Science Center at San Antonio, San Antonio, USA; 3 San Antonio Cardiovascular Proteomics Center, University of Texas Health Science Center at San Antonio, San Antonio, USA; 4 Barshop Institute for Longevity and Aging Studies, University of Texas Health Science Center at San Antonio, San Antonio, USA

## Abstract

**Background:**

About 6 million Americans suffer from heart failure and 70% of heart failure cases are caused by myocardial infarction (MI). Following myocardial infarction, increased cytokines induce two major types of macrophages: classically activated macrophages which contribute to extracellular matrix destruction and alternatively activated macrophages which contribute to extracellular matrix construction. Though experimental results have shown the transitions between these two types of macrophages, little is known about the dynamic progression of macrophages activation. Therefore, the objective of this study is to analyze macrophage activation patterns post-MI.

**Results:**

We have collected experimental data from adult C57 mice and built a framework to represent the regulatory relationships among cytokines and macrophages. A set of differential equations were established to characterize the regulatory relationships for macrophage activation in the left ventricle post-MI based on the physical chemistry laws. We further validated the mathematical model by comparing our computational results with experimental results reported in the literature. By applying Lyaponuv stability analysis, the established mathematical model demonstrated global stability in homeostasis situation and bounded response to myocardial infarction.

**Conclusions:**

We have established and validated a mathematical model for macrophage activation post-MI. The stability analysis provided a possible strategy to intervene the balance of classically and alternatively activated macrophages in this study. The results will lay a strong foundation to understand the mechanisms of left ventricular remodelling post-MI.

## Background

Myocardial infarction is defined by pathology as myocytes necrosis and apoptosis due to prolonged ischemia. Since myocytes cannot divide and replace themselves, myocytes in the infarct area deprived of oxygen die and are replaced by a collagen scar. There is a series of cellular and molecular activities respond to MI in the myocardium. Myocytes apoptosis appears in the first 6 to 8 hours post-MI, and necrosis occurs in 12 hrs to 4 days post-MI [1]. Necrosis of myocytes results in significantly elevated interlukin-1 (IL-1), tumour necrotic factor-*β *(TNF-*β*), IL-10, and monocyte chemotactic protein-1 (MCP-1) levels. MCP-1 is a strong chemoattractants that recruit and confine monocytes to the injury site. It's been reported that over 95% of monocytes differentiate to macrophages [2]. There are two major types of macrophages post-MI: classically activated macrophages (M1) and alternatively activated macrophages (M2). Porcheray has reported a switch between M1 and M2 macrophages with *in vitro *stimuli including IL-4, IL-10, and TNF-*β *[3]. In addition, biomarkers of M1 and M2 macrophages show a temporal *in vivo *transition [3]. Since M1 and M2 macrophages are responsible for extracellular matrix (ECM) destruction and construction, respectively, the transition and dynamic balance between two macrophage phenotypes might lead to the balance between ECM destruction and construction, and thus determine the ECM remodeling post-MI.[4] Therefore, characterizing macrophage activation pattern is essential to better understand the ECM remodeling post-MI.

A large amount of experimental research has been conducted to elucidate the underlying mechanisms of macrophage activation, and an abundant accumulation of experimental results define on macrophage responses to different stimuli. There is a need, however, to systemically analyze the accumulated data and integrate the results into a framework that will allow a more complete understanding. To address this need, several mathematical models have been established to characterize the effects of macrophages on wound healing, inflammatory responses, and collagen synthesis post-MI [5-9]. However, most models do not consider the effect of macrophage activation patterns and ignore the differences between macrophage phenotypes. Therefore, the aim of this study was to establish and validate a set of ordinary differential equations to characterize macrophage activation patterns post-MI. Since our mathematical model was established based on *in vivo *and *in vitro *experimental results, all parameters in the model were determined by the averages of the experimental data.

## Results

We have collected experimental data from adult C57 mice and built a framework to represent the regulatory relationship among cytokines and macrophages. Based on this framework, we established a set of nonlinear differential equations to characterize the regulatory relationship for macrophage activation in the left ventricle post-myocardial infarction using physical chemistry laws. Our framework and the mathematical model were established based on the following three assumptions.

1) All monocytes that migrate to the infarct region are differentiated to unactivated macrophages [10].

2) All activated macrophages are differentiated from unactivated macrophage since previous studies have shown that <5% of macrophages undergo mitotic division [11,12].

3) All parameters and coefficients in this model are constant.

### Framework of regulatory relationship for macrophage activation

**Figure 1:**
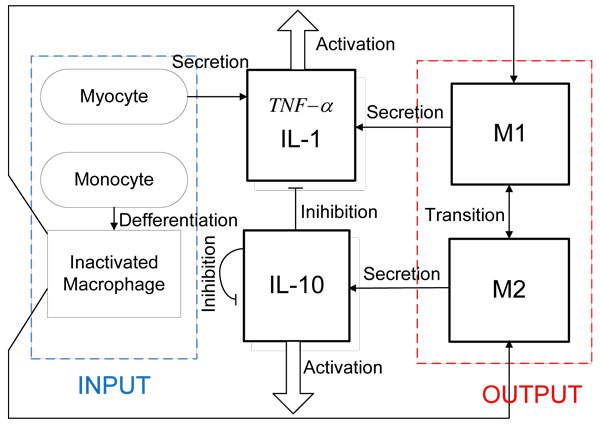
**A framework representing regulatory relationship macrophage activation post-MI**. Myocytes and monocytes are two inputs as shown in the blue dotted box. The red dotted box denotes outputs of the system, including M1 and M2 densities. Secretion function is denoted by thick arrow, activation and differentiation are denoted by thin arrow. The regulatory relationship shown in the framework was determined by published results [3,13-17].

In this framework, myocytes and monocytes were considered as inputs to the system. Cellular densities of M1 and M2 macrophages were considered as the outputs of the system. We chose IL-1, IL-10, and TNF-*α *as three molecules which regulate macrophage activations in this mathematical model since they were well-recognized as stimuli for macrophage activation [13-15]. M1 macrophages and myocytes secrete IL-1 and TNF-*α*. M2 macrophages secrete IL-10 [3]. Further, TNF-*α *and IL-1 promote M1 activation and IL_10 promotes M2 activation [16]. IL-10 inhibits TNF-*α*, IL-1, and itself [17]. The input-output and regulating relationship were shown in Figure 1.

### Input to the framework

**Figure 2:**
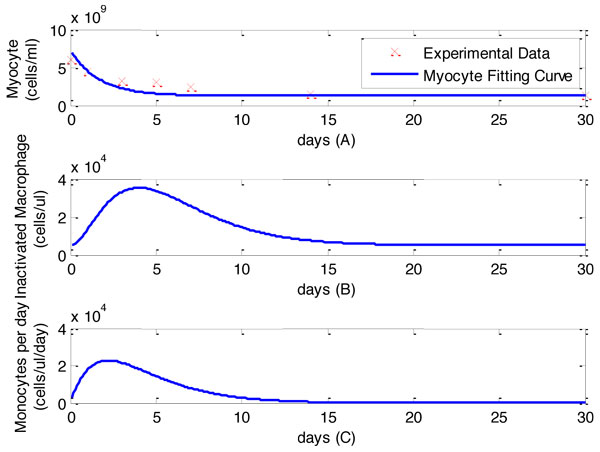
**Temporal profiles of monocytes and myocytes**. The wall thickness of left ventricle and observed a monotone decrease of wall thickness at day 1, 3, 5, 7 post-MI, suggesting the same trend of myocytes density based on the experimental results [18]. Temporal profiles of myocytes and the monotone progression trend are shown in Figure 2(A). We established the temporal profiles of monocytes based on our own experimental measurement on total macrophage density and the control value of macrophage density in health adult mice is 5 × 10^3^cells/ml. Temporal profiles of monocytes are shown in Figure 2(B). The estimation of monocytes for each day are shown in Figure 2(C).

Temporal profiles of monocytes and myocytes densities were used as inputs to our mathematical framework (Figure 1). Myocytes density in healthy adult mice was 6 × 10^9 ^cells/ml as an initial value. Myocytes numbers monotonically decreases post-MI and is directly associated with LV wall thickness. We have measured the LV wall thickness at days 0, 1, 3, 5, and 7 post-MI. The temporal profile of myocytes was determined by combining the initial value and the monotone progression trend (the crosses) as shown in Figure 2(A).

Macrophages density in the left ventricle of healthy adult mice is 2000 cells/ml, which will be used as initial values of unactivated macrophage density in this study [18,19]. Yang et al has measured temporal profiles of macrophages post-MI in mice at days 1, 2, 4, 7, 14, 21, and 28 [20]. The temporal profile of macrophages was obtained by fitting the experimental results as a continuous function as shown in Figure 2(B).

Based on our assumptions 1 and 2, all macrophages were differentiated from monocytes and emigrated from infarct area to the lymph node system. The estimation of unactivated macrophage based on the experimental results [18] is shown as follows,

(1) $$M_{un}\left(t\right)=M_{un}\left(0\right)e^{-\mu t}+\int_{0}^{t}e^{-\mu \left(t-s\right)}Mds,$$

where *M *denotes the differentiation rates of monocytes, *M_un _*denotes the unactivated macrophage density, and *μ *denotes the emigration rate of inactivated macrophage. Based on the temporal profile of unactivated macrophage, the monocytes differentiation rate can be estimated as shown in Figure 2(C).

### Mathematical model for macrophage activation

The mathematical model of macrophage activations is a set of nonlinear differential equations represented by cellular densities (cell number/ml) of *M_un_*, M1 and M2, and concentrations (pg/ml) of chemical factors such as IL-1, IL-10, and TNF-*α*. Cellular densities were determined by the difference between immigration and emigration rates. Concentrations of chemical factors were determined by the balance between their synthesis and degradation rates. The established mathematical model can be written as

(2) $$\frac{dM_{un}}{dt}=\overset{differentiation}{\overset{︷}{M}}\overset{activated\;to\;M1}{\overset{︷}{-k_{2}M_{un}\frac{IL_{1}}{IL_{1}+c_{IL1}}-k_{3}M_{un}\frac{T_{\alpha }}{T_{\alpha }+c_{T_{\alpha }}}}}-\overset{activated\;to\;M2}{\overset{︷}{k_{4}M_{un}\frac{IL_{10}}{IL_{10}+c_{IL10}}}}-\overset{emigration}{\overset{︷}{\mu M_{un}}},$$

(3) $$\frac{dM_{1}}{dt}=\overset{activation\;effects\;by\;IL_{1}\;and\;TNF_{\alpha }}{\overset{︷}{k_{2}M_{un}\frac{IL_{1}}{IL_{1}+c_{IL1}}+k_{3}M_{un}\frac{T_{\alpha }}{T_{\alpha }+c_{T_{\alpha }}}}}+\overset{transation\;between\;M1\;and\;M2}{\overset{︷}{{k^{\prime }}_{1}M_{2}-k_{1}M_{1}}}-\overset{emigration}{\overset{︷}{\mu M_{1}}},$$

(4) $$\frac{dM_{2}}{dt}=\overset{activation\;effects\;by\;IL_{10}}{\overset{︷}{k_{4}M_{un}\frac{IL_{10}}{IL_{10}+c_{IL10}}}}+\overset{transation\;between\;M1\;and\;M2}{\overset{︷}{k_{1}M_{1}-{k^{\prime }}_{1}M_{2}}}-\overset{emigration}{\overset{︷}{\mu M_{2}}},$$

(5) $$\frac{dIL_{10}}{dt}=\overset{secretion\;by\;M2}{\overset{︷}{k_{5}M_{2}\frac{c_{1}}{c_{1}+IL_{10}}}}-\overset{degradation}{\overset{︷}{d_{IL10}I\;L_{10}}},$$

(6) $$\frac{dT_{\alpha }}{dt}=\overset{secretion\;by\;M1\;and\;myocytes}{\overset{︷}{\left(k_{6}M_{1}+\lambda Mc\right)\frac{c}{c+IL_{10}}}}-\overset{degradation}{\overset{︷}{d_{T\alpha }T_{\alpha }}},$$

(7) $$\frac{dIL_{1}}{dt}=\overset{secretion\;by\;M1\;and\;myocytes}{\overset{︷}{\left(k_{7}M_{1}+\lambda Mc\right)\frac{c}{c+IL_{10}}}}-\overset{degradation}{\overset{︷}{d_{IL1}IL_{1}}},$$

where *M_un_*, *M*_1_, *M*_2 _denote the cell densities of unactivated macrophages, M1 macrophages, and M2 macrophages, respectively. Variables *IL*_10_, *T_α_*, and *IL*_1 _denote the concentrations of IL-10, TNF-*α*, and IL-1. Variable denotes *M *the differentiation rate of monocytes and *M_c _*denotes the myocytes density. The parameters used in these equations with their biological meanings, experimental values, units, and references are listed in Additional file 1. All parameters were determined based on the published data or estimation from other mathematical models [3,6,21-28].

Equation 2 determines the density of unactivated macrophages in the infarct area. For the construction part, the unactivated macrophages are differentiated from monocytes as shown in Figure 2(C). For the destruction part, the unactivated macrophages are activated to *M*_1 _or *M*_2_. Additionally, inactivated macrophages do not die locally in the scar tissue but die out in the lymph node system [27].

Equation 3 determines the activation rate of M1 macrophages. For the construction part, IL-1 and TNF-*α *promote M1 activation. Parameters *k*_2 _and *k*_3 _denote the activation rates of M1 macrophages by IL-1 and TNF-*α*. Hill equations are used to represent the promotion effects of IL-1 and TNF-*α *and parameters *c*_*IL*1 _and $c_{T_{\alpha }}$ are the effectiveness of IL-1 and TNF-*α *promotion on M1 calculated based on the experimental results [3,29]. Steinmuller has shown the transition between M1 and M2 phenotypes *in vivo *[21]. Correspondingly, we use parameter *k*_1 _to denote the transition from M1 to M2 and parameter $k_{1}^{\prime }$ for the transition from M2 to M1 [21]. The destruction part includes emigration of macrophage (*μ*)and transition from M1 to M2 macrophages (*k*_1_) [27].

Equation 4 determines the activation rate of M2 macrophages. The construction part is denoted by activation of M2 macrophages promoted by IL-10, and transition from M1 to M2. IL-10 promotes M2 activation and this activation rate has been approximated by parameters *k*_4 _based on the experimental results [3]. The transition rate from M1 to M2 is denoted as *k*_1_. The destruction part includes emigration of M2 macrophages (*μ*) and transition from M2 to M1 macrophages ($k_{1}^{\prime }$), similarly as described in equation 3.

Equation 5 determines the secretion rate of IL-10. For construction part, IL-10 is secreted by M2 macrophages, and parameter *k*_5 _denotes the secretion rate of IL-10 by M2 macrophages [22,30]. The destruction part includes the self-inhibition and degradation of IL-10. A Hill equation is employed to represent this self-inhibition effect and parameter *c*_1 _denotes the self-inhibition effect of IL-10 post-MI [6,17]. Parameter $d_{IL_{10}}$ denotes the decay rate of IL-10 determined by its half-life time [13].

Equation 6 determines the deposition rate of TNF-*α*, which is secreted by both M1 macrophages and myocytes [6,23,28,31]. We used *in vitro *results from Meng to determine the secretion rate of TNF-*α *by M1 and secretion rate of TNF-*α *by myocytes (*λ*) is determined by Horio's experimental results [23,31]. The inhibition of IL-10 is presented by a Hill equation where parameter c represents the effectiveness of IL-10 inhibiting TNF-*α *[25]. The destruction part is denoted by the degradation of TNF-*α*. Parameter $d_{T_{\alpha }}$ is the decay rate of TNF-*α *determined by its half-life time [15,26].

Equation 7 determines the deposition rate of IL-1. IL-1 is secreted by both M1 macrophage and myocytes. Parameter *k*_7 _denotes the secretion rate of IL-1 in cultured rat cardiac myocytes [31]. The inhibition of IL-10 is presented by a Hill equation similarly as in equation 6 [25]. In the destruction part, parameter *d*_*IL*1 _represents the decay rate of IL-1 determined by its half-life time [14].

### Stability analysis

If there is no myocardial infarction, monocytes differentiation and myocytes apoptosis should be at a very low level, and the studied macrophage activation pathway should maintain homeostasis. We have calculated the equilibrium point of the system without any input and performed Lyapunov stability analysis. Our analysis showed that without any monocytes differentiation and myocytes secretion, the system would stay at the origin when *t *→ ∞.

In the case of myocardial infarction, myocytes apoptosis and necrosis triggered inflammatory responses and significant monocytes differentiation, which will drive the system to a new equilibrium point. Correspondingly, the cell densities of M1 and M2 increase post-MI. We have obtained a steady state as *E *= [20, 1200, 3500, 0.73, 1.1, 5.9] from our computational simulations. The steady states match with the experimental measurements collected from healthy adults without myocardial infarction [32]. In addition, the stable region of the established mathematical model depends on the strength of the input.

### Computational results

**Figure 3:**
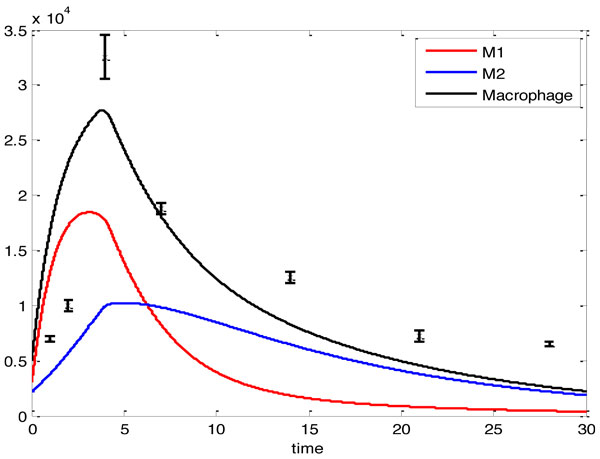
**Temporal profiles of cell densities of M1 (red line), M2 (blue line), and the total macrophage (black line) in LV 28 days post-MI**. Computational results were normalized to initial conditions and shown in solid lines. The initial cells number of unactivated macrophage is 2 × 10^3 ^cells/ml. The differentiated rate from monocytes to M1 macrophages *α *is 0.5. Then, the initial condition of macrophage densities were chosen as *M*1(0) = *M*2(0) = 0 cells/ml. Previously published experimental results were normalized to the corresponding measurements in control group and are shown as black crosses collected from C57 adult mice at days 1, 3, 5, 7, 14, 21, 28 [35]. Values are mean ± SE. All experiments were carried out in mice with MI.

**Figure 4:**
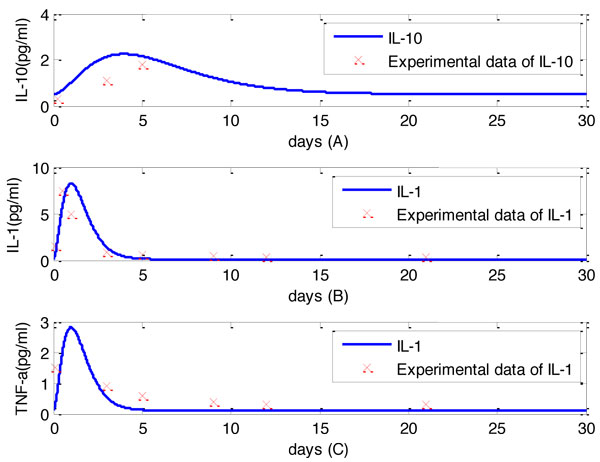
**Concentrations of IL-10, IL-1 and TNF-*α *from day 0 to day 30 post-MI**. The comparisons between the experimental measurements and temporal files were presented. In Figure 4 (A), experimental measurements of IL-10 from adult C57 mice at day 0.25, 3, 5 post-MI [34], were shown as crosses with standard deviations shown as the bar. In Figure 4 (B), experimental measurements of IL-1 from adult mice at day 0.25, 1, 3, 5, 7, 21 [36], were shown as crosses with standard deviations shown as the bar. In Figure 4 (C), experimental measurements of TNF-*α *from adult rats at day 0.25, 1, 3, 5, 7, 14, 21 [37], were shown as crosses with standard deviations shown as the bar.

Computational simulations of macrophage activation were carried out by solving the nonlinear differential equations with MATLAB. The initial conditions of unactivated, M1 and M2 macrophage densities were chosen as *M_un _*= 2000 cells/ml and *M*_1_(0) = *M*_2_(0) = 0 cells/ml. The concentrations of IL-1, TNF-*α*, and IL-10 were set as 0.1 pg/ml. The inputs of this system were shown in Figure 2. Outputs of the system, M1 and M2 densities, were shown in Figure 3. The concentrations of IL-1, IL-10 and TNF-*α *were shown in Figure 4. Our computational results were shown as solid lines while the experimental results were shown as discrete crosses in these figures [17,33,34].

Our computational results demonstrated that from days 0 to day 3 post-MI, cellular densities of the M1 phenotype increased at a faster rate than the M2 phenotype. At day 10, the M2 phenotype dominates over the M1 phenotype. This prediction agrees with the results reported by Troidl [4]. Additionally, temporal profiles of IL-1 and TNF-*α *significantly increased from days 0 to 1 post-MI in our computational simulations, which match the experimental results reported by Sumitra [33]. Comparison between the computational and experimental results demonstrates a similar trend for the temporal profiles, suggesting the effectiveness of our model.

## Discussion

This study established a mathematical model for macrophage activation in the left ventricle post-MI by combining experimental and computational approaches. This is the first mathematical model focusing on the dynamic interactions among cytokines, myocytes, monocytes, and macrophages. Computational predictions based on this mathematical model match with experimental measurements, suggesting effectiveness of the model. In addition, our stability analysis provided insight for the activation pattern of macrophages post-myocardial infarction. In our mathematical model, there are two inputs, myocytes apoptosis and monocytes differentiation. In this study, we have predicted a stable equilibrium for homeostasis, which means without myocardial infarction or following small injury stimuli, macrophage densities and concentrations of IL-1, IL-10, and TNF-α should stay at the equilibrium. After MI, the monocytes differentiated into macrophage and apoptotic myocytes secreted significant amounts of cytokines to activate the macrophages. The strength of the monocytes differentiation and myocytes apoptosis (inputs of the system) drive the system to different states while all states will be bounded due to the bounded strength of the inputs.

However, there exist some differences between computational predictions and real experimental results. To address this issue and the variation in different experiments, a stochastic parameter distribution will need to be introduced to replace the constant parameters. In addition, more detailed measurements on monocytes and myocytes and concentration temporal profiles of IL-1, IL-10 and TNF-*α *from C57 mice will be carried out in our future research, which will help to solidify our equations.

## Conclusions

Our study has established framework for macrophage activation and used ordinary differential equations to model the cellular interactions between macrophage activation types post-MI. The results on stability analysis can be used as a useful tool to predict the behaviour of biological systems.

## Methods

To incorporate the experimental data, curve fitting algorithm was applied to obtain temporal continuous density profiles of myocytes and monocytes based on discrete experimental data.

### Stability analysis of the established mathematical model

To analyse the stability of the proposed mathematical model, we have calculated the equilibrium point of the system and performed the Lyapunov stability analysis of the system.

In our mathematical model, equations (2-7) are six first-order equations with input M and *M_c_*. Now we use *x*_1_, *x*_2_, *x*_3_, *x*_4_, *x*_5_, *x*_6 _to denote *M_un_*, *M*_1_, *M*_2_, *IL*_10_, *T_α_*, *IL*_1_. We first examined the stability of the system without any input and obtained an equilibrium of $E_{1}=\left(x_{1}^{*},x_{2}^{*},x_{3}^{*},x_{4}^{*},x_{5}^{*},x_{6}^{*}\right)=\left(0,0,0,0,0,0\right)$. With the temporal input of monocytes differentiation and myocytes secretion, our computational simulation generated a steady state as $E_{2}=\left(x_{1}^{*},x_{2}^{*},x_{3}^{*},x_{4}^{*},x_{5}^{*},x_{6}^{*}\right)=\left(20,1200,3500,0.73,1.1,5.9\right)$. To further analyze the stability property of the system, we chose a positive definite Lyapunov function

(8) $$V\left(x\right)=\frac{1}{2}\sum_{i=1}^{6}x_{i}^{2}$$

and obtained its derivative as the following equation

(9) $$\begin{array}{llll} \dot{V}\left(x\right) & =\sum_{i=1}^{6}x_{i}{ẋ}_{i}\; & & \;\\ & =-\mu x_{1}^{2}-x_{1}\left[k_{2}x_{1}\frac{x_{6}}{x_{6}+c_{IL1}}+k_{3}x_{1}\frac{x_{5}}{x_{5}+c_{T_{\alpha }}}+k_{4}x_{1}\frac{x_{4}}{x_{4}+c_{IL10}}\right]\; & & \;\\ & -\left(\mu +k_{1}\right)x_{2}^{2}+k_{2}x_{2}x_{1}\frac{x_{6}}{x_{6}+c_{IL1}}+k_{3}x_{1}x_{2}\frac{x_{5}}{x_{5}+c_{T_{\alpha }}}+{k^{\prime }}_{1}x_{3}x_{2}\; & & \;\\ & -\left({k^{\prime }}_{1}+\mu \right)x_{3}^{2}+k_{1}x_{2}x_{3}+k_{4}e_{1}x_{3}\frac{x_{4}}{x_{4}+c_{IL10}}\; & & \;\\ & +k_{5}x_{3}x_{4}\frac{c_{1}}{c_{1}+x_{4}}-d_{T_{\alpha }}x_{5}^{2}+k_{6}x_{2}x_{5}\frac{c}{c+x_{4}}-d_{IL1}x_{6}^{2}+k_{7}x_{2}x_{6}\frac{c}{c+x_{4}}.\; & & \;\\ & \; & \end{array}$$

By applying the boundary of the Hill equations, we got

(10) $$\begin{array}{llll} \dot{V}\left(x\right) & \leq -\left(\mu +k_{2}+k_{3}+k_{4}\right)x_{1}^{2}-\left(\mu +k_{1}\right)x_{2}^{2}+k_{2}x_{2}x_{1}+k_{3}x_{1}x_{2}+{k^{\prime }}_{1}x_{3}x_{2}\; & & \;\\ & -\left({k^{\prime }}_{1}+\mu \right)x_{3}^{2}+k_{1}x_{2}x_{3}+k_{4}x_{1}x_{3}-d_{IL10}x_{4}^{2}+k_{5}x_{3}x_{4}-d_{T_{\alpha }}x_{5}^{2}+k_{6}x_{2}x_{5}\; & & \;\\ & -d_{IL1}x_{6}^{2}+k_{7}x_{2}x_{6}.\; & & \;\\ & \; & \end{array}$$

Applying the parameters in Table 1 to equation (10), we got

(11) $$\begin{array}{llll} \dot{V}\left(x\right) & \leq -1.6x_{1}^{2}-0.275x_{2}^{2}-0.25x_{3}^{2}-2.5x_{4}^{2}-55x_{5}^{2}-10.5x_{6}^{2}+1.1x_{1}x_{2}+0.3x_{1}x_{3}\; & & \;\\ & \;\;0.125x_{2}x_{3}+0.0005x_{3}x_{4}+0.0007x_{2}x_{5}+0.0006x_{2}x_{6}\; & & \;\\ & \leq -0.02x_{1}^{2}-0.02x_{2}^{2}-0.05x_{3}^{2}-0.5x_{4}^{2}-5x_{5}^{2}-0.5x_{6}^{2}-1.28{\left(x_{1}-0.429x_{2}\right)}^{2}\; & & \;\\ & \;-0.3{\left(x_{1}-0.5x_{3}\right)}^{2}-0.25{\left(x_{3}-0.25x_{2}\right)}^{2}-2{\left(x_{4}-0.000125x_{3}\right)}^{2}\; & & \;\\ & \;-50{\left(x_{5}-0.000007x_{2}\right)}^{2}-10{\left(x_{6}-0.00003x_{2}\right)}^{2}\; & & \;\\ & <0.\; & & \;\\ & \; & \end{array}$$

Since the derivative of Lyapunov function is negative semi-definite and the semi-definite is satisfied with all states equal to zero, the system is globally asymptotically stable without any input. Given any bounded differentiate rate of monocytes and myocytes density as input, the system will have bounded states.

## Competing interests

The authors declare that they have no competing interests.

## Authors' contributions

Y.F.J and M.L.L designed the research; Y.F.J and Y.W performed the computational analysis and simulation. Y.F.J, Y.W, T.Y, Y.M, G.V.H, M.Z, and M.L.L analyzed the results and wrote the manuscript.

## Supplementary Material

Additional file 1 **Table 1**. Pre-determined parameters from literature search. Click here for file
