# Supplementary material for: Mathematical modeling and stability analysis of macrophage activation in left ventricular remodeling post-myocardial infarction
Source: BMC Genomics. 2012 Oct 26;13(Suppl 6):S21. doi: 10.1186/1471-2164-13-S6-S21 (PMC3481436; doi:10.1186/1471-2164-13-S6-S21)
Supplement: Additional file 1 — Table 1. Pre-determined parameters from literature search. [file 1471-2164-13-S6-S21-S1.pdf]

Table 1. Pre-determined functions from literal search

Name	Biological Meaning	Value	Ref
k_1	Transition rate at which M1 is activated to become M2	0.075/day	Estimated
k_1'	Transition rate at which M2 is activated to become M1	0.05/day	[21]
k_2	Activation rate of IL-1 to activate M1.	0.1ml/pg/day	[3]
k_3	Activation rate of TNF-α to activate M1	1ml/pg/day	[3]
k_4	Activation rate of IL-10 to activate M2	0.3ml/pg/day	[3]
k_5	Secretion rate of IL-10 by M2	5×10^{-4} pg/cell/day	[22]
k_6	Secretion rate of TNF-α by M1	7×10^{-4} pg/cell/day	[39]
k_7	Secretion rate of IL-1 by M1	5×10^{-4} pg/cell/day	[23, 24]
c_1	Effectiveness of IL-10 inhibition on IL-10	100pg/ml	[39]
c	Effectiveness of IL-10 inhibition on IL-1 and TNF-α	25 pg/ml	[25]
c_{IL1}	Effectiveness of IL-1 promotion on M1	10pg/ml	[29]
c_{T_α}	Effectiveness of TNF-α promotion on M1	10pg/ml	[29]
c_{IL10}	Effectiveness of IL-10 promotion on M2	5pg/ml	[29]
d_{IL10}	Decay rate of IL-10 based on half-life time	2.5/day	[13]
$d_{T\alpha}$	Decay rate of TNF-α based on half-life time	55/day	[15, 26]
d_{IL1}	Decay rate of IL-1 based on half-life time	10.5/day	[14]
μ	M1 and M2 emigration rates	0.2/day	[40]

λ	Secretion rates of IL-1 and TNF-α by myocytes	5×10^{-6} pg/ml/cell/day	[28, 31]

*The decaying rate of chemical factors was calculated from their half-life time ($T_{1/2}$) via the equation $d = ln2/T_{1/2}$ [13-15, 26].
